# Assessment of the Railroad Transport Impact on Physical and Chemical Soil Properties: The Case Study from Zduńska Wola Karsznice Railway Junction, Central Poland

**DOI:** 10.3390/toxics9110296

**Published:** 2021-11-06

**Authors:** Ilona Tomczyk-Wydrych, Anna Świercz, Paweł Przepióra

**Affiliations:** Department of Geomorphology and Geoarchaeology, Institute of Geography and Environmental Sciences, Jan Kochanowski University, 7 Uniwersytecka St., 25-406 Kielce, Poland; swierczag@poczta.onet.pl (A.Ś.); pawelprzepiora1988@gmail.com (P.P.)

**Keywords:** potentially harmful elements (PHEs), railway area, transport pollutions

## Abstract

Contamination of the soil and water environment with harmful substances can be associated with many activities carried out on the railway. The problem is particularly relevant to liquid fuel loading and refueling facilities as well as to increased traffic at railway junctions. Studies were conducted in the area of railway junction Zduńska Wola Karsznice in central Poland (Łódź Voivodeship). Soil samples were collected from specific research points: from the inter-railway (A), 5 m from the main track (B), from the embankment—10 m from the main track (C), and from the side track (D), at the depth of 0–5 cm (1) and 20 cm (2). The following analyses were made: granulometric composition, pH in H_2_O, and percent content of carbonates (CaCO_3_). PHEs were determined in the fractions: 0.25 ≤ 0.5 mm, 0.1 ≤ 0.25 mm, and 0.05 ≤ 0.1 mm: Pb, Cd, Cr, Co, Cu, Ni, Zn, Sr by inductively coupled plasma mass spectrometry technique (ICP-MS/TOF OPTIMass 9500). The objectives of the study were (1) to assess PHEs (potentially harmful elements) contamination of the topsoil level of railway area, (2) to determine the correlation between the concentration of PHEs and the size of the fraction, and (3) to identify the areas (places) where the highest concentrations of PHEs were recorded. Based on the studied parameters, significant differentiation in soil properties of the areas in Zduńska Wola Karsznice was found. The analyses carried out showed that the accumulation of potentially harmful elements was as follows: Cu > Zn > Sr > Pb > Ni > Cr > Co > Cd. The average concentrations of Cu, Zn, Sr, Pb, Ni, Cr, Co and Cd were 216.0; 152.1; 97.8; 64.6; 15.2; 14.4; 3.1 and 0.2 mg·kg^−1^ d.w., respectively. These contaminations occur in the topsoil layer of the railway embankment, which suggests a railway transport origin. The highest concentrations of PHEs were recorded in samples collected from close to the rails (inter-railway, side track), and in the embankment (10 m from the track) in the very fine sand fraction (0.05 ≤ 0.1 mm). The high accumulation index of copper, cadmium and lead in the surface layer of soil indicate their anthropogenic origin. The results presented in the paper can be used in local planning and spatial development of this area, taking into account all future decisions about ensuring environmental protection, including groundwater and soils.

## 1. Introduction

Along with agriculture, industry, and utilities, it is transportation that contributes to a significant increase in pollution levels. Next to road transport, rail is one of the main transport means in the world. In comparison with road transport, the railroads seemed for a long time to be a relatively harmless mode of transport for the environment. This resulted in the fact that most publications were related to studies on soil contamination with potentially harmful elements (PHEs) along roads and highways in many places around the world, i.e., South Korea [1,2], Nepal [3], Tibet [4], Greece [5], Poland [6] or Thailand [7]. In the last two decades, there has been a significant increase in documentation on environmental hazards and destruction associated with rail transportation. Many studies show an increase of pollution in the immediate vicinity of transport routes, especially PHEs detected in soil and plants [8,9,10,11]. Scientists are increasingly paying more attention to the source of many contaminants from the railway. The PHEs can be connected with, i.e., abrasion of wheels and rails or even with the materials that conserve railway sleepers [12,13,14,15,16].

The increase in rail network density and traffic volume leads to higher pressure of rail transport on environmental quality, the nuisance of which depends mainly on the transport technologies used, track quality and capacity, and technical solutions used in rail vehicles. In recent years, with the increase in demand for the means of mass transportation of goods, railroads have become one of the main supply lines in the world. As a result, soil contamination along many railroad lines may have further increased. This issue has been addressed in various papers from around the world. Currently, many factors influencing the inflow of pollutants to the environment from railways are indicated, i.e., wheels abrasion [17] or sewage at railway stations [18]. The relationship between the amount of pollution and the intensity and type of transport is also clear [19,20]. However, researchers agree that rail transport is a source of numerous pollutants. Regardless of the amount of pollution, PHEs are accumulated in soil and plants, which has negative effects on the ecosystem [18,21,22,23,24,25], thereby also indicating the necessity of a way to reduce or prevent its further degradation.

The impact of rail transport on the environment is multidirectional and includes the status of soil contamination. An analysis of literature reports indicates that potentially harmful elements are among the major pollutants generated in railroad areas. The specificity of these pollutants is that they are not biodegradable and decompose to simple compounds. They have the ability to bioaccumulate and biomagnify. PHEs emitted into the environment may migrate from soils to plants, thus there is a risk of their transport to higher trophic levels [12,26].

The aim of this study was (1) to assess the PHEs contamination of the topsoil of the selected part of the Zduńska Wola Karsznice railroad, where previously, research was undertaken on a railway embankment for the first time in this place [27]. The objectives of the study were also (2) to determine the correlation between the concentration of PHEs and the size of the fraction, and (3) to identify the areas (places) where the highest concentrations of PHEs were recorded.

Moreover, the aim of this study was to compare the obtained results with tests from other sites in order to determine the probable source of potentially harmful elements origin. The following research hypothesis was evaluated: does the long-term effect of pollutants emitted by rail transport change soil parameters (including the concentration of PHEs), and if the distance from railroad track is important for topsoil contamination.

## 2. Materials and Methods

### 2.1. Study Area

**Figure 1:**
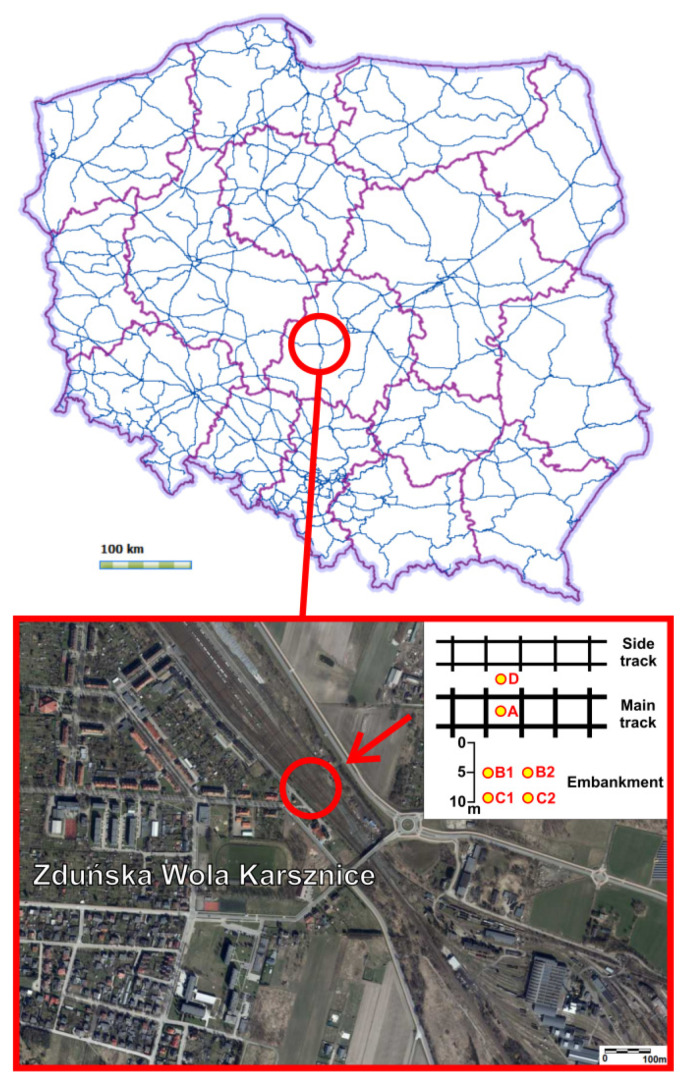
Railway network on the administrative division of Poland [34] with the location of the study area [35] with schematic sampling arrangement.

The studies on contamination of the topsoil with PHEs were conducted in the area of railroad junction Zduńska Wola Karsznice in central Poland (Łódź Voivodeship) (Figure 1). According to physico-geographical division by Kondracki [28] the area is situated in the south Wielkopolska Lowland macroregion and Łaska Upland mesoregion. The largest part of this area, included in the Łaska Upland, is covered by till, sand and gravel of glacial accumulation of the Central Poland glaciation.

The geological structure in the study area vicinity is not very diverse. The northern part of the area is built mainly by glacial sands and graves on tills, while in the southern part there are only tills. These sediments were accumulated during the Middle-Polish Glaciations [29]. In terms of soils occurring here, this area is situated on agricultural land of class I–IV [30], and usually these soils are of various agricultural suitability complexes. Very good wheat, very good rye and poor grain and fodder complexes are located closest to the examined railway section. The rest of the area is built-up (urbanized), especially to the west of the railway line with dense residential buildings. The site itself is located directly on land of agriculturally unsuitable soil complex [31]. In the Zduńska Wola Karsznice district, a very acidic (<4.5) and acidic pH (4.6–5.5) of the soils is predominant (37 and 43% of the district’s area, respectively) [32].

The railroad junction in Zduńska Wola Karsznice is located in the south-eastern part of the town (51°34′35.47″ N, 19°00′36.74″ E), 187 m above sea level (Figure 1). The 2.6 m wide railroad sleeper (inter-railway width 1.6 m) is made of treated wood. The railroad junction in Zduńska Wola Karsznice functions as a junction for freight trains travelling in the direction Wroclaw–Silesia–Central Poland. Railroad junction Zduńska Wola Karsznice is regarded as one of the biggest junctions in the country.

### 2.2. Historical News of Zduńska Wola Karsznice Railway

The construction of railroads connecting Silesia with ports was subject to economic and political factors. After the First World War, the export of coal and coke was crucial to the Polish economy. It was then decided to build two first-class, normal-track lines for public use. Despite the incomplete execution of works, in 1933 the last section of the Karsznice–Inowrocław main line was put into service. The entire line was launched on 1 August 1935, while the final completion of works took place in the spring of 1938. The railroad line was electrified between 1965 and 1969. In the initial period of the line’s operation, 12 pairs of freight trains per day were accompanied by one pair of long-distance passenger trains from Tarnowskie Góry to Gdynia, two on the Katowice–Gdynia route, two local trains from Karsznice to Inowrocław and to Częstochowa, and a connecting train from Zduńska Wola to Zduńska Wola Karsznice. In 1939 two pairs of passenger trains Gdynia–Częstochowa, Gdynia–Katowice and 10 pairs of connecting trains Zduńska Wola–Zduńska Wola Karsznice operated. Thanks to the station’s and main line’s expansion during the occupation, including the construction of the second track, the line’s capacity increased from 21 pairs of trains per day to 43 pairs in 1944. In 1950, seven pairs of passenger trains operated from Karsznice in the southern direction, while only four pairs operated in the northern direction. Sixteen shuttle trains were in use between South Karsznice and Zduńska Wola. Freight traffic reached its peak and the line’s capacity limit in 1945–1950. The 1970s saw the greatest development of the Karsznice junction. The capacity increased thanks to the line’s electrification. In 1976, 79 regular and 25 additional trains could arrive at Karsznice. Passenger train services were limited to a few pairs of passenger trains and a few, mostly seasonal, long-distance trains. With the changes in the political system, the decline of transport on the Polish State Railways (PKP) network began. In 1990, there were 64 regular and 39 additional trains in one direction, 62 regular and 49 additional in the other direction. Until the mid-1990s the number of trains in passenger traffic did not change much. Long-distance trainsets began to be reduced after 2000. Finally, passenger trains between Katowice and Inowrocław were liquidated at the end of 2008, and replaced by shortened-route connections at the end of 2010. Seasonal trains from Silesia to the seaside and passenger traffic from Zduńska Wola to Częstochowa were then abandoned. Currently, passenger traffic on the coal main line in the region of Zduńska Wola is only occasional [33].

### 2.3. Soil Sampling and Chemical Analysis

The study was conducted once in May 2013 during the preparation of the master’s dissertation [27]. Soil samples for the study were collected from four locations: inter-railway (A), 5 m from the main track (B), from the embankment—10 m from the main track (C), from the side track (D). Mineral samples were collected from two depths: 0–5 cm (1) and 20 cm (2) (Figure 1). An average of about 1 kg of soil was collected from each site.

The obtained soil material was dried at room temperature and analyzed in the Environmental Research Laboratory of the Department of Environmental Protection and Management, Jan Kochanowski University in Kielce.

The analysis of soil samples was conducted using the following methods: granulometric composition—by sieve method, pH in H_2_O—by potentiometric method, percent carbonate content (CaCO_3_)—by Scheibler method. PHEs determination was carried out in the fraction of 0.25 ≤ 0.5 mm (medium sand), 0.1 ≤ 0.25 mm (fine sand) and 0.05 ≤ 0.1 mm (very fine sand) (according to Polish Society of Soil Sciences, 2008) [36]. Sample masses of 0.1 g of soil from each fraction was weighed for mineralization in Multiwave TM 3000Anton Paar mineralizer. For this purpose, a sample containing 0.1 g was weighed and mineralized with nitric acid V (Suprapur Merck) and hydrogen peroxide in a ratio of 2.5:1 (microwave power: 1400 W, temperature: 2000 °C, time: 40 min). Certified reference materials were used for evaluating the accuracy attained. The reaction vessel was placed in a rotor which, when closed, was located in a Multiwave 3000 oven. The sample was heated by microwave radiation. Measurement data were continuously transmitted on a display. During the decomposition process, the integrated cooling system generated a flow of cooling air between the reaction vessels and their covers, thus protecting the rotor from excessive thermal load. The blanks and duplicate measurements were performed for quality control. The samples were rinsed with deionized water and the contents of potentially harmful elements (PHEs): Pb, Cd, Cr, Co, Cu, Ni, Zn, Sr were determined in the filtrate using the inductively coupled plasma mass spectrometer time-of-flight (ICP-MS/TOF) OPTIMass 9500 (GBC Scientific Equipment, Melbourne, Australia). In order to control the quality of obtained results, certified reference materials such as ERM-CA713 were used. Limit of detection (LOD) and limit of quantification (LOQ) were found between LOD: 0.05 (Cd, Cr), 0.15 (Cu, Ni, Zn), 0.5 (Pb) mg·kg^−1^ d.w.; LOQ: 0.1 (Cd, Cr), 0.2 (Ni), 0.3 (Zn), 0.5 (Cu), 1.0 (Pb) mg·kg^−1^ d.w., respectively. Precision and accuracy were controlled using certified reference materials with the same matrices for all analysts.

### 2.4. Statistical Analysis

Statistical analysis of the data was conducted using Microsoft Office Excel software (range, mean, standard deviation) (Table 1).

Based on the mean geochemical background values developed by Czarnowska [37], the PHEs accumulation index (AI) in the soil was calculated. Accumulation index is calculated using the equation:

$$\mathrm{AI}=\frac{C_{i}}{B_{i}}$$

where C_i_ is the geometric mean content of individual metals (mg·kg^−1^ d.w.); B_i_ is the geochemical background value of the elements (mg·kg^−1^ d.w.).

Taking into account the values of the geochemical background provided by the author for the examined elements, the average values for the whole of Poland were adopted: Pb 9.8 mg·kg^−1^ d.w., Cd 0.18 mg·kg^−1^ d.w., Cr 27.0 mg·kg^−1^ d.w., Co 4.0 mg·kg^−1^ d.w., Cu 7.1 mg·kg^−1^ d.w., Ni 10.2 mg·kg^−1^ d.w., Zn 30.0 mg·kg^−1^ d.w. [37].

## 3. Results

### 3.1. Physicochemical Properties of Soil Samples

**Figure 2:**
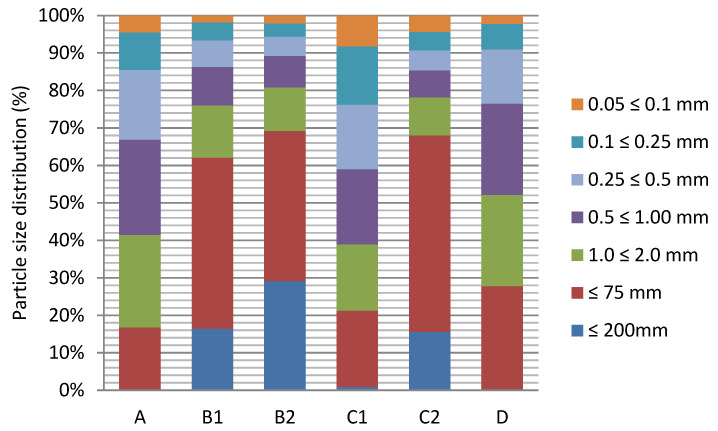
Particle size distribution (%) depending on the sampling location: A (inter-railway, depth 20 cm), B1 (5 m from the main track, depth 0–5 cm), B2 (5 m from the main track, depth 20 cm), C1 (embankment, 10 m from the main track, depth 0–5 cm), C2 (embankment, 10 m from the main track, depth 20 cm), D (side track, depth 20 cm).

The granulometric analysis showed that the grain size composition of investigated samples varied from one test point to another (according to Polish Society of Soil Sciences, PTG, 2008) [36]. From the analysis of the graphs, it was concluded that in Zduńska Wola Karsznice the majority of samples were particles larger than 2 mm (rock fraction, gravel fraction and very coarse sand fraction). Most of the samples showed enrichment in skeletal parts (Figure 2).

**Figure 3:**
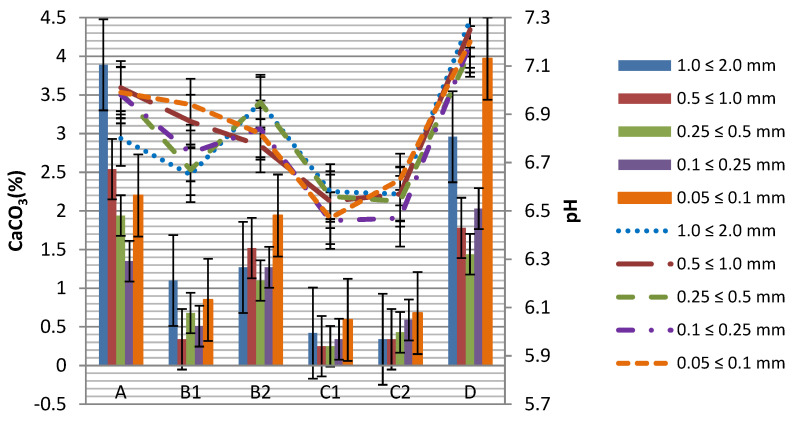
CaCO_3_ content (%) and pH value in different fractions 1.0 ≤ 2.0; 0.5 ≤ 1.0; 0.25 ≤ 0.5; 0.1 ≤ 0.25; 0.05 ≤ 0.1 (mm) depending on the sampling location with error bars. Designations: A (inter-railway, depth 20 cm), B1 (5 m from the main track, depth 0–5 cm), B2 (5 m from the main track, depth 20 cm), C1 (embankment, 10 m from the main track, depth 0–5 cm), C2 (embankment, 10 m from the main track, depth 20 cm), D (side track, depth 20 cm).

The pH values in H_2_O ranged from 6.46 (slightly acidic) to 7.29 (slightly basic). The lowest values were found in samples collected from the embankment located 10 m from the main track (6.63–6.46). The highest values were found in soils collected from the side track (7.16–7.29) and the inter-railway (6.8–7.01). The coarse sand fraction (0.5 ≤ 1.0 mm) and the very fine sand fraction (0.05 ≤ 0.1 mm) had the highest mean pH values (6.84) while the fine sand fraction (0.1 ≤ 0.25 mm) had the lowest mean pH value (6.78) (Figure 3).

The pH is a major factor affecting the solubility of metal compounds in the environment. For example, the transfer of weighting coefficient between the sorption and desorption processes of metal ions and H^+^ ions depends on pH. The literature shows that PHEs in neutral and alkaline soils migrate to a small extent to the lower layers of the soil profile due to, among other causes, limited solubility. Generally, solubility of PHEs compounds is low in neutral and alkaline pH, but much higher in acidic pH. Strong acidification of soils may contribute to the release of PHEs bound with minerals, as well as with oxides, e.g., Mn, Fe, and Al. The most mobile is Cd, while the least soluble are Pb, Cr and Hg [38]. Other metals, i.e., Zn or Cu, show increased solubility also at alkaline pH. With increasing pH, the bioavailability of metals decreases. Soils characterized by a high sorption capacity in relation to positive metal ions and containing a large amount of organic matter, show the potential to bind PHEs and retain them in the surface layer [26]. Due to the fact that the studied soils were characterized by a slightly acidic and slightly alkaline pH, the pH was not an element that significantly affected the increased mobility of metals in analyzed soils.

In the soils of the studied railroad junction, the fraction of very coarse sand (1.0 ≤ 2.0 mm) had the highest CaCO_3_ content (3.89%), and the fraction of very fine sand (0.05 ≤ 0.1 mm) 3.97%, while the fraction of medium sand (0.25 ≤ 0.5 mm) 0.25% and coarse sand (0.5 ≤ 1.0 mm) (0.25%) had the lowest content (Figure 3).

### 3.2. Levels of PHEs

The study shows that the highest concentrations of elements occurred in samples from the inter-railway (A) and the side track (D). An increase in pollutant concentrations was observed in the embankment 10 m from the track (C), which may be due to the close proximity to the road. The lowest element concentrations were recorded 5 m from the main track (B).

**Figure 4:**
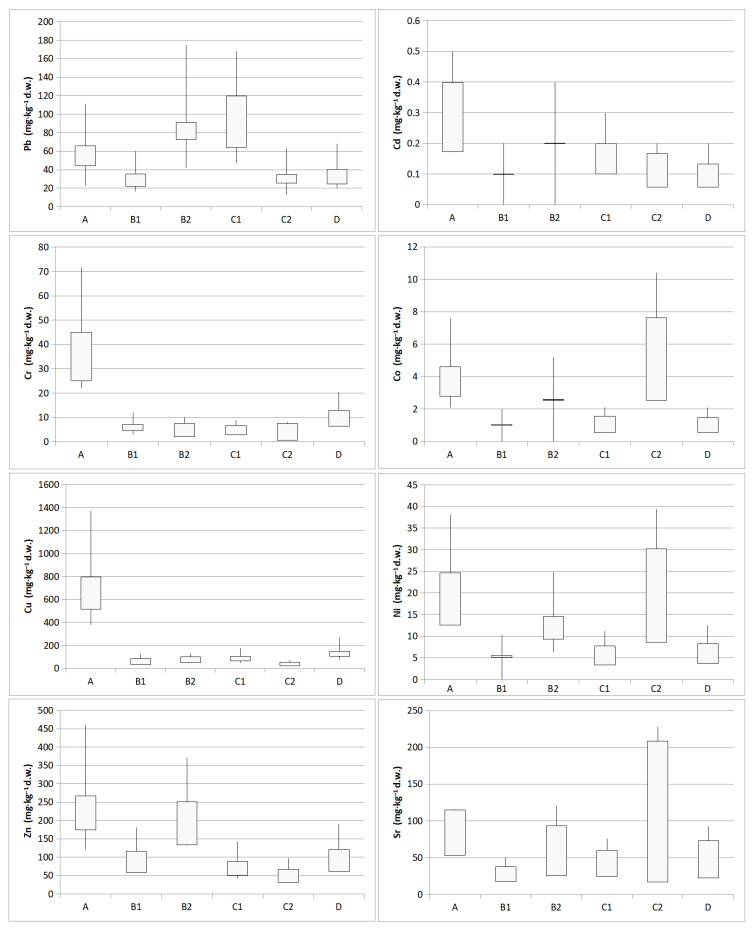
Potentially harmful elements (PHEs) content (mg·kg^−1^ d.w.) (boxplots with min., max., mean, standard deviation) according to sampling location: A (inter-railway, depth 20 cm), B1 (5 m from the main track, depth 0–5 cm), B2 (5 m from the main track, depth 20 cm), C1 (embankment, 10 m from the main track, depth 0–5 cm), C2 (embankment, 10 m from the main track, depth 20 cm), D (side track, depth 20 cm) and fraction diameter (0.05 ≤ 0.1 mm; 0.1 ≤ 0.25 mm; 0.25 ≤ 0.5 mm).

The average concentration of Pb was 64.6 mg·kg^−1^ d.w. (12.4–174.6 mg·kg^−1^ d.w.). The highest Pb concentrations were found in samples A, B2 and C1, while the lowest concentrations were found in C2. The highest concentration of Cd was found in samples A (0.5 mg·kg^−1^ d.w.), while the lowest contents were recorded in B1 and B2 (0.01 mg·kg^−1^ d.w.). Higher cadmium values were found in the 0.05 ≤ 0.1 mm fraction. The mean Cr concentration was equal to 14.5 mg·kg^−1^ d.w., the maximum, 71.8 mg·kg^−1^ d.w., was recorded in samples A, while the minimum was 3.0 mg·kg^−1^ d.w. in B1. The Co content ranged from 0.01 (B1, B2) to 10.4 mg·kg^−1^ d.w. (C2). Concentrations were comparatively low at the other sites. In the topsoil, the mean copper content was 216.2 mg·kg^−1^ d.w., the maximum 1371.9 mg·kg^−1^ d.w. (A), while the minimum was 31.0 mg·kg^−1^ d.w. (C2). The highest concentrations of Cu were recorded in the inter-railway, and the values decreased with distance from the track. The mean Ni concentration was equal to 15.2 mg·kg^−1^ d.w., with a maximum of 39.4 mg·kg^−1^ d.w., recorded in C2, and was most likely due to proximity to the road. A similar value to the maximum equal to 38.1 mg·kg^−1^ d.w. was also recorded in A. The mean Zn content was 146.3 mg·kg^−1^ d.w. with highly significant variation in results between sites. The minimum and maximum values were 34.7 mg·kg^−1^ d.w. and 460.6 mg·kg^−1^ d.w., respectively. The highest concentration of zinc in the substrate was found in samples A as well as B1 and B2. The Sr content ranged from 18.1 (B1) to 227.7 mg·kg^−1^ d.w. (C2), with a mean of 97.8 mg·kg^−1^ d.w. High concentrations were also recorded in the inter-railway (167.0 mg·kg^−1^ d.w.) (Figure 4).

**Figure 5:**
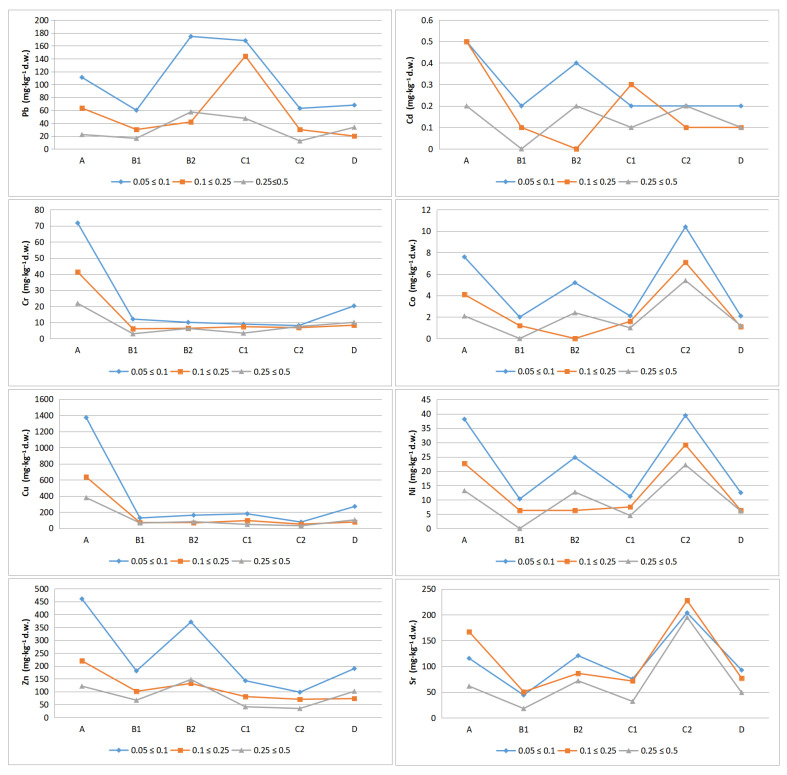
Potentially harmful elements (PHEs) content (mg·kg^−1^ d.w.) in different grain size fractions (mm) according to sampling location: A (inter-railway, depth 20 cm), B1 (5 m from the main track, depth 0–5 cm), B2 (5 m from the main track, depth 20 cm), C1 (embankment, 10 m from the main track, depth 0–5 cm), C2 (embankment, 10 m from the main track, depth 20 cm), D (side track, depth 20 cm).

Based on the study, PHEs concentrations were found to be higher in the 0.05 ≤ 0.1 mm fractions and lower in the 0.25 ≤ 0.5 mm fractions. The differences between 0.05 ≤ 0.1 mm and 0.25 ≤ 0.5 mm fractions are statistically significant (Figure 5).

Based on the mean geochemical background values developed by Czarnowska [37], an index of PHEs accumulation (AI) in the soil was calculated (Table 2). The accumulation index values for Pb, Cd, Cr, Co, Cu, Ni, and Zn ranged from 0.28 (for Cr) to 97.46 (for Cu). It should be noted that the highest accumulation index values of AI = 97.46 for copper; AI = 1.89 for cadmium; AI = 7.69 for zinc; AI = 1.49 for chromium were found in the samples from the inter-railway, the highest accumulation index of AI = 7.64 for lead was found in the samples collected 5 m from the track, and AI = 1.84 for cobalt and AI = 2.89 for nickel were found in the samples collected 10 m from the track. The mean accumulation index values in the investigated soils were arranged in the following series Cu > Zn > Pb > Ni > Cd > Co > Cr.

## 4. Discussion

The presence of PHEs in the soil around the railway is a potential threat to the natural environment. The content of metals in soils varies widely. Metals getting into soil may undergo various transformations, ranging from deposition in the form of insoluble compounds with a relatively low impact on plants and microorganisms, to occurrence in a very active ionized form. They may also form chelate connections with humic substances, which provide protection against the toxic effects of a metal ion [38,39]. Liu et al. [40] argue that high organic matter content may contribute to PHEs retention in the immediate vicinity of railroad areas.

According to Kabata-Pendias [26], the limiting content of potentially harmful elements (PHEs) in soils containing only anthropogenic pollutants is for Cd 1, Cu 25, Ni 50, Pb 70, Cr 100, and Zn 150 mg·kg^−1^. Comparing the obtained results with the limiting values of PHEs accumulated in anthropogenic soils according to Kabata-Pendias [26], it was established that mean concentrations of PHEs in samples from Zduńska Wola Karsznice were exceeded for Cu (at each sampling point), Zn (inter-railway, 5 m from the track) and Pb (10 m from the track).

The analysis of PHEs content in the topsoil of railway areas shows certain regularities in terms of their occurrence and fraction size. Representative studies that are consistent with the conclusions of this study are presented in Table 3. These studies showed that railways have significant influence on PHEs concentrations in surrounding areas, the content of several metals decreased with increasing distance from the railroad, showed increasing trend with increasing operation time of railways, and the concentration of several PHEs increased with the size of the fraction decreases.

In Zduńska Wola Karsznice the highest concentrations of PHEs are found in samples taken in close proximity to the rails, which may be the result of the impact of rail transport. In the operational sphere, trains are susceptible to destructive environmental influences (i.e., atmospheric factors causing corrosion and damage due to lightning, ultraviolet radiation and temperature changes; human activity). All of these factors contribute to the formation of specific wastes that lead to soil contamination. These include: damaged and worn out elements of the vehicle; polluting substances accumulated on the vehicle; waste created in the process of disinfecting compartments and toilets and in the process of heating, ventilating, cleaning; used technical liquids (oils, greases, hydraulic liquids) and in case of freight cars—the remains of transported loads (e.g., wood, cement, coal, liquid chemical products); sludge from domestic and sanitary wastewater (organic and inorganic particles, detergents, soaps, paper, disinfectants, fecal matter); and vapors of solvents, sulfuric and oxalic acid emitted into the atmosphere. Generally, the waste contains steel (Fe, C), non-ferrous metals (Al, Mg, Ti, Cu, Zn, Ni, Pb, Cd), rubber, plastics, chemicals, and glass. Oil and hydraulic fluids leaking from shock absorbers, discs, buffer bushings, draw hook guides and grease (Ca, Cu, Al, Li) found on the current collectors of electric locomotives pulling wagons also contribute to soil pollution [8,40,43,44]. The content of PHEs is higher in the area of the rolling stock cleaning bay and the railroad siding, while the areas of goods handling station and the platform were less contaminated. Malawska et al. [8] and Wiłkomirski et al. [45] found high contents of iron, cobalt, zinc and chromium in samples collected from the rolling stock cleaning bay and high contents of Cu, Mn and Zn at different parts of the railroad junctions. Comparing the scientists’ data with the results from Zduńska Wola Karsznice, it should be stated that the content of the mentioned elements was also at the highest level. Antuniassi et al. [46] also showed that herbicides used for conservation purposes on railroad lands contribute to soil degradation.

Taking into account the current Regulation of Minister of Environment on the method of assessing the pollution of the Earth’s surface of 1 September 2016 (Journal of Laws 2016, item 1395), it was shown that the concentration of Cu was exceeded in samples collected from the inter-railway (A) (>600 mg·kg^−1^ d.w.) [47]. The study conducted on the world’s highest railroad line, Qinghai-Tibet, found that the concentrations of Cu, Zn and Cd in the samples from the embankment were seven times higher than the geochemical background of study area [4]. According to Moczarski [44], and Wiłkomirski et al. [43] copper oxides formed from oxidized power collectors, catenary lines and intensive operation of pantographs by rail vehicles.

It can be expected that the increased level of lead in samples taken 5 and 10 m from the tracks may be the result of the impact of road transport, as shown in the studies by Liu et al. [40]. Similar observations were made by Vaiškūnaitė and Jasiūnienė [15], who found that the highest concentrations of Pb and Cd were recorded at a distance of 5.0 m from railroad sleepers in the upper (up to 10 cm) layer of soil.

The increase in lead and chromium concentration in the topsoil adversely affects the microfauna and microflora. As a result of reducing the enzymatic activity of soil microorganisms, the process of decomposition of organic matter slows down, which in turn leads to soil degradation [26].

The study does not support the hypothesis that Pb concentration increases with the depth of soil profile. The study of Łukasiewicz [48] showed that lead was present in the upper part of the profile at 6 of 21 sites, while already deeper at 19 sites. This is not consistent with the results presented in this paper.

Cadmium was characterized by the lowest concentration in the studied soil samples. Concentration of this element does not show any change depending on the depth of sampling. It is accumulated both in shallower and deeper soil profiles. The investigated soil samples contain high concentrations of manganese, which sorbs cadmium, thus decreasing its mobility in the environment and availability to the flora. The content of Zn does not show a strong correlation with the depth of sampling, which confirms that Zn is a common element and its content in soil strongly depends on the content in the source rock [49]. However, the presence of lead is largely the result of anthropogenic activity rather than the result of source rock.

It should be noted that the highest concentrations of Pb, Cd, Cr, Co, Cu, Ni, and Zn occur in 0.05 ≤ 0.1 mm fractions. As the fraction size increases, the concentration of PHEs decreases. In the smallest fractions of the tested soils (≤0.1 mm), the content of lead, cadmium, chromium, cobalt, copper, nickel and zinc exceeded 168.1; 0.5; 71.8; 10.4; 1371.9; 39.4; and 460.6 mg·kg^−1^ d.w., respectively. The richest in metals are fine-grained fractions separated from the soils of A (Cd, Cr, Cu, Zn), B2 (Pb), C2 (Co, Ni).

The concentration of PHEs in soil also depends on the duration of exposure to the contamination. Despite the fact that the authors did not attempt to confirm this thesis in these studies, these issues are often discussed in the world literature. According to Chen et al. [10], soils were contaminated with Cd and Pb, and their maximum content was determined in samples from railroad areas with the longest operating time. The concentration levels of Zn, Cu and Fe in soil samples are not affected by anthropogenic activities.

## 5. Conclusions

This study examined the physical and chemical properties in surface soils from Zduńska Wola Karsznice railway junction. The study found that the pH values of investigated soils ranged from 6.47 (slightly acidic) to 7.29 (slightly alkaline). With increasing distance from the rails, the pH value decreased. Through analysis of accumulation index (AI) the mean values of PHEs in the investigated soils were arranged in the following series Cu > Cd > Pb > Zn > Ni > Co > Cr. The high accumulation index of copper, cadmium and lead in the surface layer of soil indicate their anthropogenic origin. The average concentrations of particular metals in the soil of railroad junction in Zduńska Wola Karsznice were as follows: Cu > Zn > Sr > Pb > Ni > Cr > Co > Cd. Higher PHEs contents were found in the very fine sand fraction (0.05 ≤ 0.1 mm).

The highest concentrations of potentially harmful elements were recorded in samples collected from close to the rails (inter-railway, side track), and in the embankment (10 m from the track). The high level of PHEs contamination of soils in railroad areas can have a destabilizing effect on ecosystems. The discovery of increased concentrations of PHEs in railway junctions is of great importance, not only cognitive, but also practical, e.g., in the process of remediation of these areas. The authors are well aware of the limitations resulting from a low number of soil samples taken and plan to increase the number of soil samples and to extend the scope of methods. In addition, the authors recommend the determination of PHEs in the smallest fractions during continuous monitoring studies (0.05 ≤ 0.1 mm). The highest concentration of metals was recorded in these fractions. These findings should contribute to assessing sources for further migration of PHEs into the groundwater, crops, and finally, humans. This will allow the visualization of the potential risk to living organisms. Moreover, zoning plans should consider these results when planning new agricultural lands and single family houses.

## Figures and Tables

**Table 1:** Metals contamination (mg·kg^−1^ d.w.) depending on the sampling location: A (inter-railway, depth 20 cm), B1 (5 m from the main track, depth 0–5 cm), B2 (5 m from the main track, depth 20 cm), C1 (embankment, 10 m from the main track, depth 0–5 cm), C2 (embankment, 10 m from the main track, depth 20 cm), D (side track, depth 20 cm).

Metal	Property	Sampling Location					
		A	B1	B2	C1	C2	D
Pb	Range	22.3–111.1	16.4–60.0	41.9–174.6	47.4–168.1	12.4–63.0	19.9–68.0
	Mean	65.6	35.5	91.3	119.9	35.1	40.5
	SD	44.44	22.29	72.55	63.89	25.68	24.76
Cd	Range	0.2–0.5	0.01–0.2	0.01–0.4	0.1–0.3	0.1–0.2	0.1–0.2
	Mean	0.4	0.1	0.2	0.2	0.1	0.1
	SD	0.17	0.11	0.23	0.12	0.06	0.06
Cr	Range	21.9–71.8	3.0–12.1	6.3–10.1	3.4–9.0	6.8–8.1	8.3–20.3
	Mean	45.0	7.1	7.6	6.6	7.5	12.9
	SD	25.15	4.63	2.16	2.88	0.65	6.47
Co	Range	2.1–7.6	0.01–2.0	0.01–5.2	1.0–2.1	5.4–10.4	1.1–2.1
	Mean	4.6	1.1	2.5	1.6	7.6	1.5
	SD	2.78	1.01	2.60	0.55	2.54	0.55
Cu	Range	379.4–1371.9	65.3–127.2	65.7–131.9	47.0–178.9	31.0–76.1	76.4–270.5
	Mean	795.9	87.7	102.8	106.9	52.6	150.1
	SD	515.12	34.34	51.74	66.77	22.61	105.16
Ni	Range	13.2–38.1	0.01–10.3	6.3–24.8	4.5–11.2	22.2–39.4	6.1–12.5
	Mean	24.7	5.5	14.6	7.7	30.3	8.3
	SD	12.56	5.19	9.39	3.36	8.65	3.64
Zn	Range	121.4–460.6	66.9–180.7	132.0–370.8	41.3–142.7	34.7–98.0	73.2–189.8
	Mean	267.3	116.3	251.4	88.3	67.7	121.5
	SD	174.51	58.35	133.67	51.10	31.74	60.82
Sr	Range	61.5–115.5	18.1–50.5	71.8–120.7	32.0–75.5	195.1–227.7	49.2–92.7
	Mean	114.7	37.6	92.9	59.6	208.9	72.9
	SD	52.75	17.18	25.12	24.02	16.88	22.02

**Table 2:** Contamination degree based on accumulation index (AI).

Sampling Location	Index of PHEs Accumulation						
	Pb	Cd	Cr	Co	Cu	Ni	Zn
Inter-railway	5.5	1.89	1.49	1.00	97.46	2.21	7.69
5 m from the main track	7.64	0.53	0.27	0.12	13.39	1.23	6.43
10 m from the main track	2.92	0.88	0.28	1.84	6.95	2.89	2.07
Side track	3.65	0.66	0.44	0.35	18.37	0.77	3.74

**Table 3:** Representative research similar to this study.

Study Area	Main Conclusion	References
Suining Railway Station, Suining, Sichuan Province, China	The concentrations of Cd, Pb showed increasing trend with increasing operation time of railways.	[10]
Railway that connects České Budějovice and Brno cities	The highest copper content was observed in soils taken close to the railroad.	[13]
Railway tracks on the territory of Srem, Serbia	The concentration values of Cu, Ni, Cd, Pb in samples collected from up to 1 km from the railroad line were higher than in samples collected from >1 km.	[14]
Suining, Sichuan, China	The railway transportation caused heavy metal pollution and the degree was Mn > Cd > Cu > Zn > Pb.	[20]
Railroad areas in Tarnowskie Góry	The level of PHEs contamination near railroad sites decreased with increasing distance from the rails.	[41]
Outlet roads in Poznań, Polska	The highest metal concentrations were observed in the smallest fraction (<0.063 mm), which were up to four times higher than those in sand fractions.	[42]

## Data Availability

Not applicable.
